# Breastfeeding Duration and Child Development

**DOI:** 10.1001/jamanetworkopen.2025.1540

**Published:** 2025-03-24

**Authors:** Inbal Goldshtein, Yair Sadaka, Guy Amit, Nitsa Kasir, Thomas Bourgeron, Varun Warrier, Pinchas Akiva, Meytal Avgil Tsadok, Deena R. Zimmerman

**Affiliations:** 1 KI Research Institute, Kfar Malal, Israel; 2 Neuro-Developmental Research Center, Mental Health Institute, Be’er-Sheva, Israel; 3 Faculty of Health Sciences, Ben-Gurion University of the Negev, Be’er-Sheva, Israel; 4 National Insurance Institute, Jerusalem, Israel; 5 Human Genetics and Cognitive Functions Unit, Institut Pasteur, Paris, France; 6 Department of Psychiatry, University of Cambridge, United Kingdom; 7 TIMNA Initiative, Big Data Platform, Israel Ministry of Health, Jerusalem, Israel; 8 Maternal Child and Adolescent Department, Public Health Directorate, Ministry of Health, Jerusalem, Israel

## Abstract

**Importance:**

Detecting and addressing potentially modifiable factors associated with healthy development is key to optimizing a child’s potential. When investigating the outcomes of child development, it is important to account for disparities in feeding practices and avoid confounding bias.

**Objectives:**

To estimate the independent association between breastfeeding and attainment of developmental milestones or neurodevelopmental conditions.

**Design, Setting, and Participants:**

This retrospective cohort study used data from a national network for routine child development surveillance in Israel linked with national social insurance financial entitlements for neurodevelopmental deficiencies. Participants were children born between January 2014 and December 2020 after at least 35 weeks’ gestation without severe morbidity and with at least 1 follow-up surveillance visit at 2 to 3 years of age. Outcome data were collected in March 2023.

**Exposures:**

Duration and exclusivity of breastfeeding in infancy.

**Main Outcomes and Measures:**

The primary outcomes were delays in attainment of developmental milestones and diagnosis of prespecified neurodevelopmental conditions. Multivariable regression, matching, and within-family analyses were used to estimate adjusted odds ratios (AORs) after accounting for potential confounding factors related to the child (gestational age, birth weight, multiple gestation, and child order in the family) and mother (age, socioeconomic status, educational level, marital status, employment, nationality, and postpartum depression).

**Results:**

Of 570 532 children (291 953 [51.2%] male), 20 642 (3.6%) were preterm, 38 499 (6.7%) were small for gestational age, and 297 571 (52.1%) were breastfed for at least 6 months (123 984 [41.7%] were exclusively breastfed). Children who were breastfed for at least 6 months exhibited fewer delays in attaining language and social or motor developmental milestones compared with children exposed to less than 6 months of breastfeeding (AOR, 0.73 [95% CI, 0.71-0.76] for exclusive breastfeeding; AOR, 0.86 [95% CI, 0.83-0.88] for nonexclusive breastfeeding). Among 37 704 sibling pairs, children who were breastfed for at least 6 months were less likely to demonstrate milestone attainment delays (OR, 0.91 [95% CI, 0.86-0.97]) or be diagnosed with neurodevelopmental conditions (OR, 0.73 [95% CI, 0.66-0.82]) compared with their sibling with less than 6 months of breastfeeding or no breastfeeding.

**Conclusions and Relevance:**

In this cohort study, exclusive or longer duration of breastfeeding was associated with reduced odds of developmental delays and language or social neurodevelopmental conditions. These findings may guide parents, caregivers, and public health initiatives in promoting early child development.

## Key Points

**Question:**

Is breastfeeding associated with improved neurodevelopment outcomes after adequate control for potential confounders?

**Findings:**

In this cohort study of 570 532 children in Israel, longer and exclusive breastfeeding were independently associated with lower odds of developmental delays after adjusting and matching for key confounders. Among 37 704 sibling pairs, children who were breastfed for at least 6 months were less likely to demonstrate milestone attainment delays or neurodevelopmental deficiencies compared with their sibling with less than 6 months of or no breastfeeding.

**Meaning:**

These findings support current infant feeding recommendations.

## Teaser

This cohort study assesses the association between breastfeeding duration and exclusivity and attainment of developmental milestones or neurodevelopmental conditions among children aged 2 to 3 years.

## Introduction

Early childhood is an important time to promote healthy physical and cognitive development by addressing potentially modifiable factors, such as nutrition. The World Health Organization (WHO) recommends exclusive breastfeeding for the first 6 months of life, followed by continued breastfeeding alongside healthy complementary foods for up to 2 years or beyond.^1^

Previous studies demonstrating a positive correlation between breastfeeding and measures of cognitive development were limited by confounding bias.^2,3^ Socioeconomic status or maternal educational level are associated both with breastfeeding and with neurodevelopmental outcomes, yet they are not routinely documented.^4^ Complicated births, prematurity, and newborn illnesses often influence early breast attachment as well as long-term developmental state. Neonates born before full term have a higher risk of developmental delays^5,6,7^ and a greater potential benefit from breastfeeding.^8^ However, the concern for growth among neonates born preterm (<37 weeks’ gestation) or small for gestational age (SGA) may lead to reduced breastfeeding and prioritize commercial formula supplementation. Careful control for confounding is crucial^2,9,10^ and requires comprehensive datasets.

Additionally, many studies classify breastfeeding as a dichotomy, that is, either breast milk or formula feeding within a prespecified time window, whereas in practice, many infants receive both. Therefore, it is of interest to investigate the duration of any breastfeeding, not necessarily exclusive breastfeeding. The current study aimed to estimate the association between breastfeeding and child development in both early-term to postterm (37-42 weeks’ gestation) and late-preterm (35-36 weeks’ gestation) children using the linkage of 2 unique data sources: routine developmental surveillance visits in national mother-child health clinics (MCHCs), and national insurance disability entitlements records.

## Methods

The Soroka Medical Center institutional review board approved this cohort study with an informed consent waiver due to data deidentification. The data were extracted using TIMNA, a national research platform established by the Israeli Ministry of Health to enable big data studies that combine deidentified health data from multiple organizations. The study followed the Strengthening the Reporting of Observational Studies in Epidemiology (STROBE) reporting guideline for cohort studies.

### Setting

The MCHCs in Israel provide free pediatric preventive services for children from birth to 6 years of age by public health trained nurses and physicians. They cover over 70% of the national pediatric population. The services include vaccinations, early detection of health problems, routine monitoring of growth and development, and education promoting healthy lifestyle, such as nutrition, hygiene, and safety. The recommended visit times are after birth and at 1, 2, 4, 6, 9, 12, 18, 24, 36, 48, and 60 months.

The MCHC centralized electronic health record system includes maternal covariates, such as age, birth country, nationality, educational level, employment, marital status, and Edinburgh Postnatal Depression Scale (EPDS) score, and the child’s information, such as sex, birth-related covariates (gestational age, Apgar score, birth weight, birth type, multiple gestation, and length of hospital stay), clinical data collected during routine visits, nutrition, and developmental status. The database includes biosocial risk, defined by the Ministry of Social Affairs and Social Services^11^ (risks of physical health, residence, emotional environment, and violence).

The second source of data was the National Insurance Institute of Israel (NII), a governmental agency in charge of social security and welfare, which provides an allowance for entitled families with a child with disability. Not all diagnosed cases of a certain condition are entitled to NII benefits—only those involving a substantial functional disorder requiring support and supervision. Consequently, the data identifies actual disabilities rather than diagnoses. For example, eligibility based on attention-deficit/hyperactivity disorder (ADHD) is granted only to children with severe ADHD who study in a special education framework and receive pharmaceutical treatment.^12^

### Study Population

The study included children born between January 2014 and December 2020 who had at least 1 follow-up surveillance visit at 2 to 3 years of age, were born after at least 35 weeks’ gestation, did not have severe illness, and were discharged from the hospital within 5 days of birth. The rationale for these restrictions was to reduce confounding by poor health. Children born earlier than 35 weeks’ gestation are more likely to receive breathing support or require other medical equipment and experience stressful events in the neonatal intensive care unit, which hamper milk supply and successful breastfeeding. The focus on children who were early-term to postterm and late-preterm neonates covered most children (eFigure 1 in Supplement 1) while minimizing bias.

Severe illness was defined based on the NII records. It included vision loss or blindness, hearing loss, Down syndrome, severe chronic disease requiring full supervision, severe medical impairment requiring full supervision, catheterization, missing 2 limbs, gastrostomy, prolonged feeding and nutrition disorders, or medical home care.

Ethnicity was ascertained by self-report and assessed to understand population diversity and to plan tailored strategies to cultivate various subgroups. Ethnic groups included Christian Arab, Druse, Jewish, Muslim Arab, Muslim Bedouin, and other, which included Circassian, other Christian, and other Muslim.

### Exposures

In each visit, the nurses interviewed the mothers regarding the child’s current nutrition and documented the findings in a dedicated and mandatory electronic form. The mothers were specifically asked whether they breastfed, and if they ceased, the cessation age was documented.

Breastfeeding exposure was expressed in 2 different manners: categorical and continuous. The categorical representation was an ordered variable of less than 6 months of breastfeeding (including no breastfeeding and nonexclusive breastfeeding for <6 months), nonexclusive breastfeeding for at least 6 months, and exclusive breastfeeding for at least 6 months. The 6-month cutoff was chosen for comparability with the WHO recommendations.^1^ The continuous representation was defined by the duration of any breastfeeding (in months).

### Outcomes

Developmental outcomes included (1) delayed attainment of milestones and (2) a diagnosis of a neurodevelopmental condition (NDC), each captured by a different data source. Outcome data collection was conducted in March 2023.

Milestone delay was based on MCHC visit records. Child development was measured by the Tipat-Halav Israeli Surveillance standardized scale; the validation is described elsewhere.^13,14^ In each visit, a nurse records the child’s attainment of a set of age-appropriate milestones. When a child does not attain a milestone that is attained by 90% of the population at that age, the nurse refers the parents to further medical attention and support as needed. Nonattainment of a milestone on time is not diagnostic or synonymous with developmental delay; however, it is a proxy linked with developmental vulnerabilities. Milestone delay was defined based on 6 milestones assessed at 24 to 36 months of age, split into 2 sets: 3 language or social milestones and 3 gross or fine motor milestones (eTable 1 in Supplement 1). For each set, milestone delay was defined as nonattainment of at least 2 (of 3) milestones when assessed after the 90% normative passing age (according to the Tipat-Halav Israeli Surveillance scale). Overall milestone delay was defined as delay in either the language or social set or the motor set.

An NDC was defined based on NII entitlements. Language or social NDCs included autism, pervasive developmental disorder, or assisted communication; ADHD; and severe behavioral disorder. Motor NDCs included cerebral palsy, paralysis, or malfunction of 2 limbs. The following records were considered unclassified NDC: developmental NDC, low developmental quotient (<62), or epilepsy entitling NII benefits. Overall NDC was defined as language or social NDC, motor NDC, or unclassified NDC. Due to the low rate of motor difficulties and undetermined NDC, the multivariable models for these outcomes could not control for all confounders, and they were examined as exploratory outcomes.

### Confounding Variables

The following covariates were used as control variables: socioeconomic status, gestational age, birth type (spontaneous, cesarean delivery, or instrumental), multiple birth vs singleton, postpartum depression risk (captured by the EPDS score), biosocial risk, maternal marital status, country of origin, educational level, and SGA^15^ according to validated Dolberg curves.^16^ Socioeconomic status was determined based on the geographic statistic area of the attended MCHC with use of information from the Israel Central Bureau of Statistics.^17^ Additional variables were used for adjustment only in models for common outcomes, as they presented weaker potential to confound the association (based on a data-driven approach of standardized mean differences [SMDs] <0.1 between exposure groups). These additional variables included maternal age and employment, newborn position, birth year, and child sex.

Parental dedication or engagement was not directly measured in this study. As partial proxies, we examined compliance to routine MCHC visits and iron or vitamin D administration. In addition, we conducted within-family comparisons, assuming that siblings are often exposed to similar levels of parental dedication.

### Statistical Analysis

Three analytical approaches were applied. The first approach was a multivariable logistic regression model aimed to estimate adjusted odds ratios (AORs) for the associations between breastfeeding and developmental outcomes. Restricted cubic splines were used to reflect the nonlinear association between the outcome and the covariates of gestational age, socioeconomic status, and breastfeeding duration. The number of knots was selected based on the Akaike information criterion. To avoid overfitting, in the case of binary covariates, the model included only covariates with at least 100 children in each category. Interaction terms were added to examine whether the association between breastfeeding and outcomes varied by socioeconomic status or by prematurity.

The second approach was to select a covariate-matched sample by matching all covariates that showed an SMD greater than 0.1. The third approach was a within-family design. This design is commonly used to overcome unobservable confounders by conditioning out maternal and environmental factors that are likely shared between siblings (particularly parental dedication, which is difficult to measure). This analysis included pairs of siblings born with a similar gestational age yet exposed to different breastfeeding patterns. The feasibility of sibling analyses depends on a sufficient sample of eligible families. Due to the rarity of 2 premature children in the same family, the preterm siblings subpopulation was primarily composed of twins, who are rarely fed differently; the subset of preterm siblings with discordant feeding and developmental outcomes was too small to allow statistical inference. Consequently, the within-family analysis was conducted only among early-term to postterm infants. The OR for developmental outcomes after controlling for family was calculated by the McNemar test for paired data and by multivariable conditional logistic regression to adjust for residual confounding between siblings.

Analyses were performed in R, version 4.2.2 (R Project for Statistical Computing).^18^ A 2-sided *P* < .05 was considered statistically significant.

## Results

### Population Description

A total of 690 358 children born between 2014 and 2020 after at least 35 weeks’ gestation visited an MCHC. After excluding children with illness or long birth hospitalization (9%) and those with missing data (9%; socioeconomic status, birth weight, or number of children per pregnancy), 570 532 children remained (eFigure 1 in Supplement 1). Of these children, 48.8% were female and 51.2% were male. A total of 1.5% were Christian Arab; 2.0%, Druse; 56.4%, Jewish; 23.5%, Muslim Arab; 1.3%, Muslim Bedouin; and 2.1%, other ethnicity. In the entire cohort, 96.4% of children were early term to postterm, 3.6% were preterm, 6.7% were small for gestational age, and 52.2% were breastfed for at least 6 months. A total of 37 450 (6.5%) had milestone attainment delay (5.8% language or social, 1.2% motor), and 14 446 (2.5%) had an NDC (2.3% language or social, 0.03% motor, and 0.1% undetermined).

eFigure 2 in Supplement 1 depicts breastfeeding persistence rates. Overall, 123 984 children in the cohort (21.7%) breastfed for at least 6 months exclusively, 173 587 (30.4%) breastfed for at least 6 months nonexclusively, and 272 961 (47.8%) were not breastfed or were breastfed for less than 6 months. Longer breastfeeding duration was more common among infants born at a later gestational age, singleton births, nonfirstborn, spontaneous vaginal delivery, married mothers, higher educational level, lower socioeconomic status, no postpartum depression, and no biosocial risks (Table 1).

**Table 1.** Baseline Characteristics of Study Population

Characteristic	Participants, No. (%)		
	Breastfeeding <6 mo (n = 272 961)	Nonexclusive breastfeeding ≥6 mo (n = 173 587)	Exclusive breastfeeding ≥6 mo (n = 123 984)
Breastfeeding duration, median (IQR), mo	1 (0-3)	10 (7-15)	14 (12-20)
Multiple pregnancy	9612 (3.5)	3824 (2.2)	542 (0.4)
Gestational age group			
Moderate or late preterm	12 709 (4.7)	5481 (3.2)	2452 (2.0)
Early term to postterm	260 252 (95.3)	168 106 (96.8)	121 532 (98.0)
Gestational age, median (IQR), wk	39 (38-40)	39 (38-40)	39 (39-40)
Small for gestational age	20 899 (7.7)	11 232 (6.5)	6368 (5.1)
Firstborn	179 168 (65.6)	108 795 (62.7)	70 328 (56.7)
Maternal educational level			
Academic	77 253 (28.3)	57 813 (33.3)	43 281 (34.9)
High school	89 581 (32.8)	40 366 (23.3)	22 840 (18.4)
Tertiary	22 001 (8.1)	17 622 (10.2)	13 856 (11.2)
Elementary	5991 (2.2)	3962 (2.3)	1727 (1.4)
Missing	78 135 (28.6)	53 824 (31.0)	42 280 (34.1)
Ethnic group			
Christian Arab	4877 (1.8)	2448 (1.4)	1301 (1.0)
Druse	6177 (2.3)	4144 (2.4)	1360 (1.1)
Jewish	153 066 (56.1)	90 021 (51.9)	78 602 (63.4)
Muslim Arab	65 060 (23.8)	48 203 (27.8)	21 001 (16.9)
Muslim Bedouin	4534 (1.7)	2306 (1.3)	668 (0.5)
Other^a^	5549 (2.0)	3397 (2.0)	2805 (2.3)
Missing	33 698 (12.3)	23 068 (13.3)	18 247 (14.7)
Sex			
Female	132 771 (48.6)	83 694 (48.2)	62 114 (50.1)
Male	140 190 (51.4)	89 893 (51.8)	61 870 (49.9)
Maternal birth country			
Israel	221 478 (81.1)	139 426 (80.3)	97 434 (78.6)
Former Soviet Union	16 865 (6.2)	8117 (4.7)	7487 (6.0)
Europe	3960 (1.5)	2197 (1.3)	2245 (1.8)
America	2056 (0.8)	2434 (1.4)	3081 (2.5)
Ethiopia	3913 (1.4)	3531 (2.0)	749 (0.6)
Other	1528 (0.6)	1434 (0.8)	812 (0.7)
Missing	23 161 (8.5)	16 448 (9.5)	12 176 (9.8)
Maternal employment			
Working	120 963 (44.3)	75 138 (43.3)	55 043 (44.4)
Not working	58 511 (21.4)	37 177 (21.4)	23 031 (18.6)
Student	10 833 (4.0)	7894 (4.5)	5906 (4.8)
Missing	82 654 (30.3)	53 378 (30.7)	40 004 (32.3)
Socioeconomic status score, median (IQR)^b^	5 (3-6)	4 (3-6)	5 (3-6)
Any comorbidity	61 042 (22.4)	34 172 (19.7)	21 679 (17.5)
Any biosocial risk	47 566 (17.4)	22 404 (12.9)	12 154 (9.8)
Any vision disorder	14 678 (5.4)	8640 (5.0)	5181 (4.2)
Marital status			
Married	232 337 (85.1)	150 564 (86.7)	107 007 (86.3)
Not married	17 907 (6.6)	7191 (4.1)	4960 (4.0)
Missing	22 717 (8.3)	15 832 (9.1)	12 017 (9.7)
Maternal age group, y			
≤20	1201 (0.4)	546 (0.3)	351 (0.3)
21-40	237 801 (87.1)	148 828 (85.7)	107 194 (86.5)
>40	24 633 (9.0)	17 606 (10.1)	10 474 (8.4)
Missing	9326 (3.4)	6607 (3.8)	5965 (4.8)
Maternal age, median (IQR), y	29 (25-33)	29 (25-34)	29 (25-33)
Newborn position			
Head	226 670 (83.0)	148 639 (85.6)	106 274 (85.7)
Breech	9579 (3.5)	4542 (2.6)	2144 (1.7)
Other	2898 (1.1)	1676 (1.0)	961 (0.8)
Missing	33 814 (12.4)	18 730 (10.8)	14 605 (11.8)
Birth type			
Spontaneous	188 863 (69.2)	132 843 (76.5)	99 867 (80.5)
Cesarean delivery	53 012 (19.4)	24 147 (13.9)	12 342 (10.0)
Instrumental	15 965 (5.8)	9029 (5.2)	5697 (4.6)
Missing	15 121 (5.5)	7568 (4.4)	6078 (4.9)
EPDS score^c^			
<10	197 135 (72.2)	129 484 (74.6)	93 362 (75.3)
≥10	9230 (3.4)	5159 (3.0)	2375 (1.9)
Missing	66 596 (24.4)	38 944 (22.4)	28 247 (22.8)
Birth year, median (IQR)	2017 (2015-2019)	2017 (2015-2019)	2017 (2015-2019)
Apgar score 1 min			
0-3	1128 (0.4)	571 (0.3)	409 (0.3)
4-6	3907 (1.4)	2078 (1.2)	1226 (1.0)
7-10	264 688 (97.0)	169 059 (97.4)	120 643 (97.3)
Missing	3238 (1.2)	1879 (1.1)	1706 (1.4)
Apgar score 5 min			
0-3	443 (0.2)	267 (0.2)	233 (0.2)
4-6	415 (0.2)	243 (0.1)	133 (0.1)
7-10	267 212 (97.9)	170 188 (98.0)	121 126 (97.7)
Missing	4891 (1.8)	2889 (1.7)	2492 (2.0)

Abbreviation: EPDS, Edinburgh Postnatal Depression Scale.

^a^ Other included Circassian, other Christian, and other Muslim.

^b^ Socioeconomic scores ranged from 0 to 10, with higher numbers indicating higher socioeconomic level.

^c^ EPDS scores ranged from 0 to 30, with higher numbers indicating greater likelihood of experiencing postpartum depression.

Examination of health routines indicated no association between breastfeeding and adherence to supplementation recommendations, with adherence rates of 92.4% vs 95.5% (SMD, 0.13) for vitamin D and 84.4% vs 87.2% (SMD, 0.08) for iron among breastfeeding mothers vs others. Similarly, breastfeeding was not associated with adherence to recommended visits (80.9% vs 85.8% visited after age 5 years among breastfeeding vs others; SMD, 0.14). Consequently, these variables were not included in the model as proxies for health behavior.

### Multivariable Regression

eTable 2 in Supplement 1 depicts the results of a multivariable logistic regression model with exposure split into 3 categories, with breastfeeding for less than 6 months used as the reference. Breastfeeding was associated with lower odds of milestone attainment delay for both motor and language or social milestones. The association had an AOR of 0.86 (95% CI, 0.83-0.88) for nonexclusive breastfeeding and an AOR of 0.73 (95% CI, 0.71-0.76) for exclusive breastfeeding. The odds of motor NDC did not significantly differ between breastfeeding groups.

These findings persisted when the OR was modeled as a flexible spline of breastfeeding duration (Figure). The ORs of milestone attainment delay gradually decreased with longer breastfeeding (compared with no breastfeeding), particularly in the first months of life, reaching a near plateau after 10 to 12 months (ceiling effect). There was no evidence for lower odds of motor NDC. The absolute probabilities of developmental outcomes stratified by breastfeeding exposure are depicted in eFigure 3 in Supplement 1. The odds were particularly reduced for language or social development.

**Figure.**
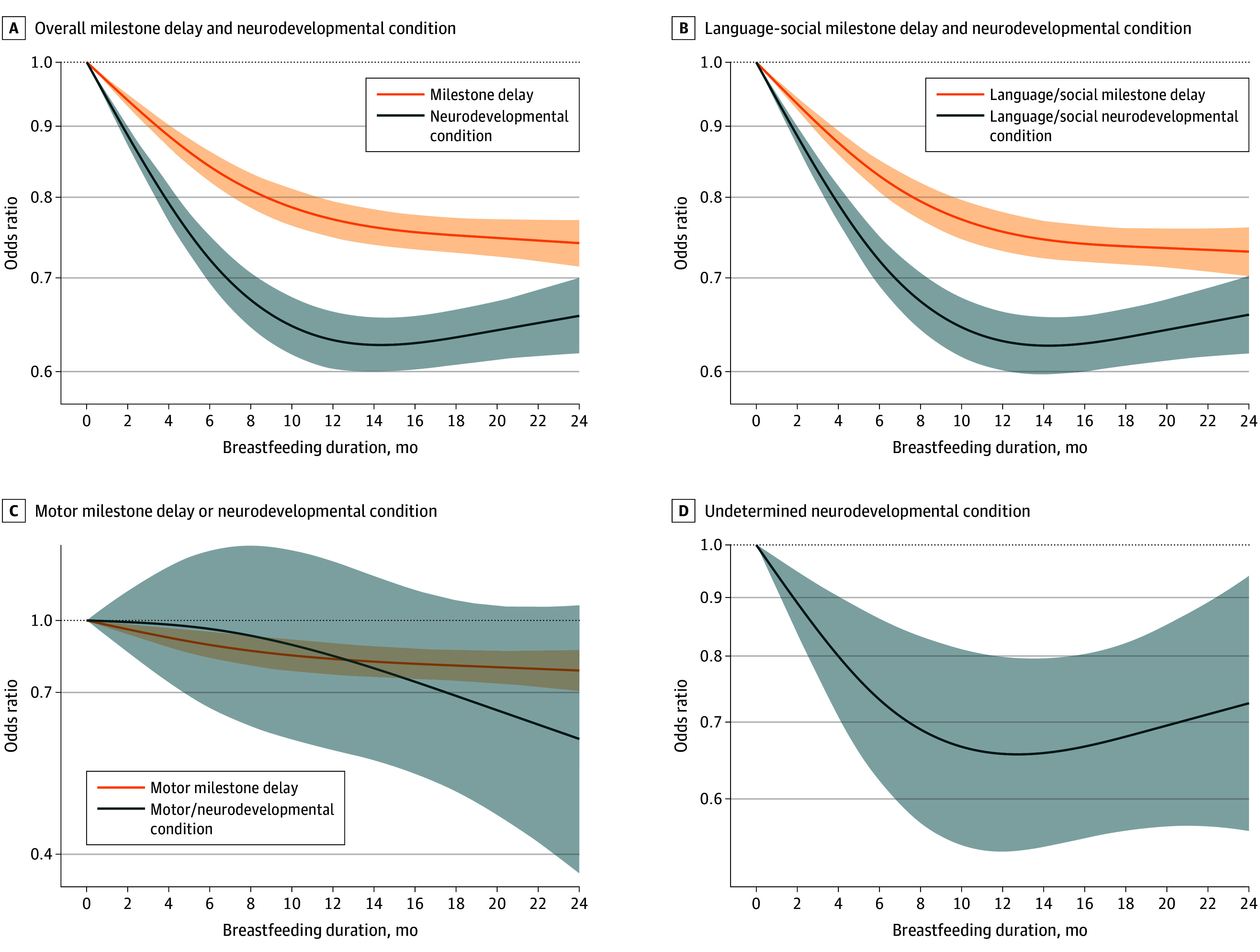
Restricted Cubic Spline of Odds Ratios for Developmental Outcomes as a Function of Breastfeeding Duration Analyses were adjusted for maternal-, birth-, and infant-related factors; 0 months was the reference. Shading indicates 95% CIs.

### Covariate Matched Sample Analysis

The matched cohort included 228 210 pairs of children exposed or not exposed to at least 6 months of breastfeeding. The cohorts were well balanced (SMD <0.1) (eTable 3 in Supplement 1) except on birth year, which was thus adjusted for within the regression model. The AOR for delay in milestone attainment was 0.83 (95% CI, 0.81-0.85), ranging between 0.82 (95% CI, 0.80-0.84) for language or social milestone delay and 0.88 (95% CI, 0.83-0.93) for motor milestone delay (Table 2). The AOR for NDC was 0.72 (95% CI, 0.69-0.75), varying between 0.72 (95% CI, 0.69-0.75) for language or social NDC, 0.74 (95% CI, 0.62-0.87) for undetermined NDC, and 0.76 (95% CI, 0.54-1.07) for motor NDC, which was not significant.

**Table 2.** Adjusted Odds Ratios for Developmental Outcomes in the Matched Cohort

Outcome, breastfeeding exposure	Adjusted odds ratio (95% CI)	*P* value
Any milestone delay		
≥6 mo	0.83 (0.81-0.85)	<.001
<6 mo	1 [Reference]	NA
Language or social milestone delay		
≥6 mo	0.82 (0.80-0.84)	<.001
<6 mo	1 [Reference]	NA
Motor milestone delay		
≥6 mo	0.88 (0.83-0.93)	<.001
<6 mo	1 [Reference]	NA
Any NDC		
≥6 mo	0.72 (0.69-0.75)	<.001
<6 mo	1 [Reference]	NA
Language or social NDC		
≥6 mo	0.72 (0.69-0.75)	<.001
<6 mo	1 [Reference]	NA
Motor NDC		
≥6 mo	0.76 (0.54-1.07)	.12
<6 mo	1 [Reference]	NA
Undetermined NDC		
≥6 mo	0.74 (0.62-0.87)	<.001
<6 mo	1 [Reference]	NA

Abbreviation: NA, not applicable; NDC, neurodevelopmental condition.

The interactions between prematurity and breastfeeding were nonsignificant (*P* value for interaction ranged between .20 and .50 for all outcomes), indicating no modification of associations by prematurity. Nonetheless, due to higher incidence of developmental outcomes among preterm children, their absolute risk difference was more pronounced compared with their early-term to postterm counterparts (Table 3).

**Table 3.** Absolute Differences of Developmental Outcomes by Breastfeeding Exposure in the Matched Cohort, Stratified by Prematurity

Outcome, breastfeeding exposure	Individuals, %	Absolute difference (95% CI)
**Any milestone delay**		
Late preterm		
≥6 mo	6.8	−1.89 (−1.99 to −1.79)
<6 mo	8.7	
Early term to postterm		
≥6 mo	5.9	−1.18 (−1.27 to −1.08)
<6 mo	7.1	
**Any neurodevelopmental condition**		
Late preterm		
≥6 mo	2.5	−1.27 (−1.33 to −1.21)
<6 mo	3.7	
Early term to postterm		
≥6 mo	2.1	−0.73 (−0.79 to −0.68)
<6 mo	2.8	

### Sibling Analysis

We detected 37 704 families with at least 2 early-term to postterm siblings with discordant exposure to breastfeeding (<6 months vs ≥6 months) and randomly sampled a pair of siblings from each family. On average, the child exposed to breastfeeding for 6 months or longer was less likely to be firstborn than the unexposed sibling (40.4% vs 47.7%; SMD, 0.15) (eTable 4 in Supplement 1) and less likely to have missing data on birth type.

There were 11% fewer pairs for which milestone delay was observed in the sibling exposed to at least 6 months of breastfeeding than pairs with delay in the unexposed sibling (2000 vs 2237; McNemar test *P* < .001). In addition, there were 25% fewer pairs in which an NDC was observed in the exposed sibling than pairs with an NDC in the unexposed sibling (621 vs 825; McNemar test *P* < .001).

Table 4 reports conditional logistic regression results among children exposed to at least 6 months of breastfeeding and unexposed children, accounting for correlation among siblings and controlling for multiple pregnancy, child order in the family, and postpartum depression. The OR was 0.91 (95% CI, 0.86-0.97) for delay in milestone attainment and 0.73 (95% CI, 0.66-0.82) for NDC. Further analyses for specific outcomes revealed significant associations with language or social milestone delay (OR, 0.93 [95% CI, 0.87-0.99]; *P* = .02), language or social NDC (OR, 0.77 [95% CI, 0.69-0.86]; *P* < .001), and motor milestone delay (OR, 0.76 [95% CI, 0.66-0.87]; *P* < .001), but there was no association for the rarer outcome of motor NDC (OR, 0.75 [95% CI, 0.38-1.47]; *P* = .40).

**Table 4.** Odds Ratios for Developmental Outcomes in the Siblings Cohort

Outcome	Odds ratio (95% CI)	*P* value
**Any milestone delay**		
Any breastfeeding		
≥6 mo	0.91 (0.86-0.97)	.003
<6 mo	1 [Reference]	NA
Exclusive vs nonexclusive breastfeeding		
≥6 mo exclusive	0.94 (0.86-1.02)	.13
≥6 mo nonexclusive	1 [Reference]	NA
**Any neurodevelopmental condition**		
Any breastfeeding		
≥6 mo	0.73 (0.66-0.82)	<.001
<6 mo	1 [Reference]	NA
Exclusive vs nonexclusive breastfeeding		
≥6 mo exclusive	0.87 (0.75-1.01)	.06
≥6 mo nonexclusive	1 [Reference]	NA

Abbreviation: NA, not applicable.

Among infants exposed to at least 6 months of breastfeeding, we detected 26 549 families with at least 1 sibling breastfed exclusively and another sibling breastfed nonexclusively during the first 6 months of life. There were no associations between exclusive breastfeeding and developmental outcomes for milestone attainment delay (OR, 0.94 [95% CI, 0.86-1.02]) or NDC (OR, 0.87 [95% CI, 0.75-1.01]).

## Discussion

The current study examined the association between breastfeeding and developmental outcomes in a large, heterogenous setting. The findings indicated a reduced likelihood for developmental delays and dysfunction among children exposed to longer breastfeeding. The ORs were slightly lower in within-family analyses than in between-family analyses yet remained statistically significant.

The association between breastfeeding duration and developmental outcomes was similar for late-preterm and early-term to postterm infants. While the ORs were similar, the absolute difference depended on the baseline rates. NDCs were more common among preterm children, yielding greater absolute benefits. Similarly, an earlier investigation showed larger cognitive benefits among infants with low birth weight compared with those with normal birth weight.^19^

Prior studies reported conflicting results. In a sample of 2734 sibling pairs, cognitive ability was significantly associated with breastfeeding,^9^ whereas an earlier, smaller study indicated no difference in intelligence.^20^ The current study relied on larger and more contemporary data compared with the aforementioned reports.

In the current study’s setting, we were unable to discern between human milk and human interaction. Skin-to-skin contact may have an independent effect. A recent trial^21^ found no developmental benefits for bottle-fed donor milk over artificial milk. However, the trial was restricted to extremely preterm children, characterized by high rates of morbidity. Moreover, pasteurized donor milk differs from pumped mother’s own milk.

Several biological mechanisms have been proposed for the differences in cognitive development. Victora et al^22^ postulated that benefits of breastfeeding for brain development may be mediated through effects on the infant microbiome. Neuroimaging studies have shown structural differences in white matter, gray matter, and neuronal connectivity.^23,24,25^

This work evaluated the potential contribution of breastfeeding to favorable development. The aim of this study was not to alter or reduce neurodivergence but rather to maximize each individual child’s abilities and minimize functional gaps, thereby promoting inclusion of neurodiversity in a world with a majority neurotypical population. We made efforts to define relevant variables while being constrained by the secondary use of data.

The WHO strongly endorses breastfeeding, yet a gap remains between feeding recommendations and actual practices. Breastfeeding is a learned behavior on the part of the mother and child and thrives best when there is adequate promotion, protection, and support. The Baby-Friendly Hospital Initiative provided effective evidence-based guidance for such conditions,^26,27^ yet its implementation was not globally widespread.^28^ Many women with intent to breastfeed discontinue as they face the challenges of balancing breastfeeding with needs of home or employment. Furthermore, the infant food industry spends $55 billion annually on promoting their products using methods that may undermine women’s confidence in their ability to successfully breastfeed.^29^ Therefore, it is important to advocate for breastfeeding-supportive parental leave and employment policies and to reduce inappropriate marketing tactics for infant foods.^30^

### Strengths and Limitations

The strengths of this study are the large nationwide population sample and data availability of critical confounders. The use of routine surveillance data reduced the risk of recall bias.

This study has limitations. The risk of confounding by infant illness was mitigated using national security entitlement records and duration of birth hospitalization, but we could not rule out the possibility that some illnesses were not fully captured. Parental engagement and dedication or paternal intelligence are unmeasured confounders, which were mitigated by the within-family design. However, the siblings analysis was not feasible among preterm children.

## Conclusions

In this cohort study, breastfeeding persistence was associated with lower incidence of developmental delays. These findings may guide parents, caregivers, and public health initiatives in promoting optimal child development.

## Notes

Supplement 1. **eTable 1.** Milestones utilized to define milestone developmental delay **eTable 2.** Odds ratios (95% CI) for developmental outcomes in the full cohort **eTable 3.** Population characteristics before and after matching, by breastfeeding exposure **eTable 4.** Population characteristics, siblings’ analysis **eFigure 1.** Study population flowchart **eFigure 2.** Histogram of breastfeeding duration (months) **eFigure 3.** Restricted cubic spline for the absolute probability of developmental outcomes as a function of breastfeeding duration

Supplement 2.
Data Sharing Statement

## References

[1] Meek JY, Noble L; Section on Breastfeeding. Policy statement: breastfeeding and the use of human milk. Pediatrics. 2022;150(1):e2022057988. doi:10.1542/peds.2022-057988 35921640

[2] Pereyra-Elías R, Quigley MA, Carson C. To what extent does confounding explain the association between breastfeeding duration and cognitive development up to age 14? findings from the UK Millennium Cohort Study. PLoS One. 2022;17(5):e0267326. doi:10.1371/journal.pone.0267326 35613097 PMC9132301

[3] Jain A, Concato J, Leventhal JM. How good is the evidence linking breastfeeding and intelligence? Pediatrics. 2002;109(6):1044-1053. doi:10.1542/peds.109.6.1044 12042541

[4] Hair AB, Patel AL, Kiechl-Kohlendorfer U, . Neurodevelopmental outcomes of extremely preterm infants fed an exclusive human milk-based diet versus a mixed human milk + bovine milk-based diet: a multi-center study. J Perinatol. 2022;42(11):1485-1488. doi:10.1038/s41372-022-01513-3 36171356 PMC9616714

[5] Hua J, Barnett AL, Lin Y, . Association of gestational age at birth with subsequent neurodevelopment in early childhood: a national retrospective cohort study in China. Front Pediatr. 2022;10:860192. doi:10.3389/fped.2022.860192 35712637 PMC9194570

[6] Dueker G, Chen J, Cowling C, Haskin B. Early developmental outcomes predicted by gestational age from 35 to 41 weeks. Early Hum Dev. 2016;103:85-90. doi:10.1016/j.earlhumdev.2016.07.006 27536852

[7] Larsen ML, Wiingreen R, Jensen A, . The effect of gestational age on major neurodevelopmental disorders in preterm infants. Pediatr Res. 2022;91(7):1906-1912. doi:10.1038/s41390-021-01710-4 34420036

[8] Belfort MB. Human milk and preterm infant brain development. Breastfeed Med. 2018;13(S1):S23-S25. doi:10.1089/bfm.2018.29079.mbb 29624427

[9] Evenhouse E, Reilly S. Improved estimates of the benefits of breastfeeding using sibling comparisons to reduce selection bias. Health Serv Res. 2005;40(6 Pt 1):1781-1802. doi:10.1111/j.1475-6773.2005.00453.x 16336548 PMC1361236

[10] Colen CG, Ramey DM. Is breast truly best? estimating the effects of breastfeeding on long-term child health and wellbeing in the United States using sibling comparisons. Soc Sci Med. 2014;109(109):55-65. doi:10.1016/j.socscimed.2014.01.027 24698713 PMC4077166

[11] Schmid H, Dolev T, Szabo-Lael R. Community-based programs for children at risk: the case of budget flexibility in Departments of Social Services in Israel. Child Youth Serv Rev. 2010;32(2):178-184. doi:10.1016/j.childyouth.2009.08.006

[12] Disabled child—benefits. State of Israel National Insurance Institute. Accessed January 7, 2024. http://www.btl.gov.il:80/English%20Homepage/Benefits/Disabledchild/Pages/default.aspx

[13] Girshovitz I, Amit G, Goldshtein I, . Increased rates of unattained developmental milestones among Israeli children between 2016 and 2020: a national report. Isr J Health Policy Res. 2023;12(1):38. doi:10.1186/s13584-023-00586-5 38129917 PMC10740256

[14] Sudry T, Zimmerman DR, Yardeni H, . Standardization of a developmental milestone scale using data from children in Israel. JAMA Netw Open. 2022;5(3):e222184. doi:10.1001/jamanetworkopen.2022.2184 35285917 PMC9907346

[15] Gutbrod T, Wolke D, Soehne B, Ohrt B, Riegel K. Effects of gestation and birth weight on the growth and development of very low birthweight small for gestational age infants: a matched group comparison. Arch Dis Child Fetal Neonatal Ed. 2000;82(3):F208-F214. doi:10.1136/fn.82.3.F208 10794788 PMC1721075

[16] Dollberg S, Haklai Z, Mimouni FB, Gorfein I, Gordon ES. Birth weight standards in the live-born population in Israel. Isr Med Assoc J. 2005;7(5):311-314. 15909464

[17] Hananel R, Fishman R, Malovicki-Yaffe N. Urban diversity and epidemic resilience: the case of the COVID-19. Cities. 2022;122:103526. doi:10.1016/j.cities.2021.103526 34908641 PMC8660207

[18] R: The R Project for Statistical Computing. Accessed June 4, 2024. https://www.r-project.org/

[19] Ip S, Chung M, Raman G, et al. Breastfeeding and maternal and infant health outcomes in developed countries. American Academy of Pediatrics Grand Rounds. 2007;18(2):15-16. PMC4781366 17764214

[20] Der G, Batty GD, Deary IJ. Effect of breast feeding on intelligence in children: prospective study, sibling pairs analysis, and meta-analysis. BMJ. 2006;333(7575):945. doi:10.1136/bmj.38978.699583.55 17020911 PMC1633819

[21] Colaizy TT, Poindexter BB, McDonald SA, ; Eunice Kennedy Shriver National Institute of Child Health and Human Development Neonatal Research Network; MILK Trial Investigators. Neurodevelopmental outcomes of extremely preterm infants fed donor milk or preterm infant formula: a randomized clinical trial. JAMA. 2024;331(7):582-591. doi:10.1001/jama.2023.27693 38497706 PMC10828950

[22] Victora CG, Bahl R, Barros AJD, ; Lancet Breastfeeding Series Group. Breastfeeding in the 21st century: epidemiology, mechanisms, and lifelong effect. Lancet. 2016;387(10017):475-490. doi:10.1016/S0140-6736(15)01024-7 26869575

[23] Kar P, Reynolds JE, Grohs MN, . Association between breastfeeding during infancy and white matter microstructure in early childhood. Neuroimage. 2021;236:118084. doi:10.1016/j.neuroimage.2021.118084 33882345

[24] Núñez C, García-Alix A, Arca G, . Breastfeeding duration is associated with larger cortical gray matter volumes in children from the ABCD study. J Child Psychol Psychiatry. 2023;64(7):1067-1079. doi:10.1111/jcpp.13790 36946606

[25] Paquette AF, Carbone BE, Vogel S, . The human milk component *myo*-inositol promotes neuronal connectivity. Proc Natl Acad Sci U S A. 2023;120(30):e2221413120. doi:10.1073/pnas.2221413120 37433002 PMC10374161

[26] Implementation guidance: protecting, promoting, and supporting breastfeeding in facilities providing maternity and newborn services: the revised Baby-Friendly Hospital Initiative 2018. World Health Organization. Accessed May 5, 2024. https://www.who.int/publications-detail-redirect/9789241513807

[27] Pérez-Escamilla R, Martinez JL, Segura-Pérez S. Impact of the Baby-friendly Hospital Initiative on breastfeeding and child health outcomes: a systematic review. Matern Child Nutr. 2016;12(3):402-417. doi:10.1111/mcn.12294 26924775 PMC6860129

[28] National implementation of the Baby-Friendly Hospital Initiative 2017. World Health Organization. Accessed May 1, 2024. https://iris.who.int/bitstream/handle/10665/255198/WHO-NMH-NHD-17.4-eng.pdf?sequence=1

[29] Lancet T; The Lancet. Unveiling the predatory tactics of the formula milk industry. Lancet. 2023;401(10375):409. doi:10.1016/S0140-6736(23)00118-6 36764311

[30] Guidance on ending the inappropriate promotion of foods for infants and young children: implementation manual. World Health Organization. Published online 2017. Accessed May 1, 2024. https://www.who.int/publications/i/item/9789241513470

