# Supplementary material for: Breastfeeding Duration and Child Development
Source: JAMA Netw Open. 2025 Mar 24;8(3):e251540. doi: 10.1001/jamanetworkopen.2025.1540 (PMC11933992; doi:10.1001/jamanetworkopen.2025.1540)
Supplement: Supplement 1. — eTable 1. Milestones utilized to define milestone developmental delay eTable 2. Odds ratios (95% CI) for developmental outcomes in the full cohort eTable 3. Population characteristics before and after matching, by breastfeeding exposure eTable 4. Population characteristics, siblings’ analysis eFigure 1. Study population flowchart eFigure 2. Histogram of breastfeeding duration (months) eFigure 3. Restricted cubic spline for the absolute probability of developmental outcomes as a function of breastfeeding duration [file jamanetwopen-e251540-s001.pdf]

Supplementary Online Content

Goldshtein I, Sadaka Y, Amit G, et al. Breastfeeding duration and child development. *JAMA Netw Open*. 2025;8(3):e251540. doi:10.1001/jamanetworkopen.2025.1540

eTable 1. Milestones utilized to define milestone developmental delay

eTable 2. Odds ratios (95% CI) for developmental outcomes in the full cohort

eTable 3. Population characteristics before and after matching, by breastfeeding exposure

eTable 4. Population characteristics, siblings' analysis

eFigure 1. Study population flow chart

eFigure 2. Histogram of breastfeeding duration (months)

eFigure 3. Restricted cubic spline for the absolute probability of developmental outcomes as a function of breastfeeding duration

This supplementary material has been provided by the authors to give readers additional information about their work.

© 2025 Goldshtein I et al. *JAMA Network Open*.

eTable 1. Milestones utilized to define milestone developmental delay

Milestone	Method of evaluation	Pass
Recognizes familiar objects and pronounces them by name (Language-social)	The toddler is asked to name a variety of items or pictures that the examiner points to in the room while asking "What is it? What is it called?"	The toddler will name several items from the familiar environment, such as: clothing, furniture, games, fruits, animals and more. Mispronunciations are acceptable.
Understands actions and speech without gestures (Language-social)	The toddler is required to understand and perform actions asked by the examiner, literally without marking with a gesture or hint, with objects from the assessment kit. For example: Show me what do we drink with or give me the cup.	The toddler understands at least 3 actions such as: give, show, put. Understands at least one preposition such as - on, within.
Participates in a dialogue (Language -Social)	It is advisable to listen during the entire visit to the conversation that develops between the mother and the toddler. It should be monitored and seen whether a dialogue develops with the adults during the evaluation, whether the child expresses feelings and desires appropriately. Does he ask situation appropriate questions like: who? what? where?	The toddler initiates a verbal dialogue with the examiner or parent in a suitable situation, expresses his desires, responds to his interlocutor's response and able to share experiences from everyday life. Please note: Reverberating words without meaning or intention of communication will not be considered as speech aimed at communication purposes and requires further evaluation.
Runs well without falling (Motor)	If possible, performed during the visit at the station. The examiner should pay attention to the child's organization before performing the task. Multiple falls requires attention and reference	The toddler runs freely and confidently, stepping on each foot fully, while paying attention to obstacles in his path and his ability to bypass them without dropping them.
Climbs up and down the stairs without an adult's assistance (Motor)	If possible, performed during the visit at the station. The child should be encouraged to go up and down the stairs with a railing that he can hold under supervision.	The toddler goes up and down the stairs with the help of a railing, without the support of an adult.
Imitates horizontal, vertical and circle lines (Motor)	The toddler sits on a chair at a small table next to the parent. The examiner or the parent hands the child a sheet of paper and a pencil, shows him how to draw horizontal, vertical and circle lines.	The toddler imitates horizontal, vertical and circle lines.

© 2025 Goldshtein I et al. *JAMA Network Open*.

eTable 2. Odds ratios (95% CI) for developmental outcomes in the full cohort

Outcome	Breastfeeding exposure	Odds Ratio (95% CI)	P-value
Any milestone delay	Exclusive 6 months	0.73 (0.71 to 0.76)	<.001
	Non-exclusive 6 months	0.86 (0.84 to 0.88)	<.001
	Less than 6 months	1 (Ref.)	
Motor milestone delay	Exclusive 6 months	0.85 (0.79 to 0.91)	<.001
	Non-exclusive 6 months	0.91 (0.86 to 0.96)	0.001
	Less than 6 months	1 (Ref.)	
Language social milestone delay	Exclusive 6 months	0.71 (0.69 to 0.73)	<.001
	Non-exclusive 6 months	0.85 (0.83 to 0.88)	<.001
	Less than 6 months	1 (Ref.)	
Any neurodevelopmental conditions	Exclusive 6 months	0.60 (0.57 to 0.63)	<.001
	Non-exclusive 6 months	0.75 (0.72 to 0.78)	<.001
	Less than 6 months	1 (Ref.)	
Motor neurodevelopmental conditions	Exclusive 6 months	1.01 (0.68 to 1.49)	0.96
	Non-exclusive 6 months	0.71 (0.50 to 1.01)	0.06
	Less than 6 months	1 (Ref.)	
Language social neurodevelopmental conditions	Exclusive 6 months	0.59 (0.56 to 0.62)	<.001
	Non-exclusive 6 months	0.75 (0.72 to 0.79)	<.001
	Less than 6 months	1 (Ref.)	
Undetermined neurodevelopmental conditions	Exclusive 6 months	0.68 (0.55 to 0.84)	<.001
	Non-exclusive 6 months	0.82 (0.69 to 0.96)	0.01
	Less than 6 months	1 (Ref.)	

© 2025 Goldshtein I et al. *JAMA Network Open*.

eTable 3. Population characteristics before and after matching, by breastfeeding exposure

The matching was exact for categorical covariates (bio-social risk, multiple pregnancy, firstborn) and caliper for continuous covariates (gestational age, socioeconomic status, birth year).

	**Pre-Matching, Children, No. (%)**			**Post-Matching, Children, No. (%)**		
	**Less than 6 months of breastfeeding**	**Breastfeeding for at least 6 months**		**Less than 6 months of breastfeeding**	**Breastfeeding for at least 6 months**	
**Characteristic, n (%)**	**N = 272,961**	**N = 297,571**	**SMD**	**N = 228,210**	**N=228,210**	**SMD**
Breastfeeding months, Median (IQR)	1 (0-3)	12 (9-18)	2.79	1 (0-3)	12 (8-18)	2.24
Multiple pregnancy	9,612 (3.5)	4,366 (1.5)	0.13	3,842 (1.7)	3,866 (1.7)	<0.001
Gestational age group						
Moderate/Late preterm	12,709 (4.7)	7,933 (2.7)	0.11	7,933 (3.5)	7,933 (3.5)	<0.001
Term	260,252 (95)	289,638 (97)		220,277 (97)	220,277 (97)	
Gestational age, Median (IQR)	39 (38-40)	39 (38-40)	0.20	39 (38-40)	39 (38-40)	0.043
Small for gestational age	20,899 (7.7)	17,600 (5.9)	0.069	16,399 (7.2)	13,806 (6.0)	0.046
Firstborn	179,168 (66)	179,123 (60)	0.11	146,494 (64)	146,446 (64)	<0.001
Maternal Education			0.3			<0.001
Academic	77,253 (28)	101,094 (34)		72,553 (32)	72,587 (32)	
High School	89,581 (33)	63,206 (21)		61,604 (27)	61,536 (27)	
Tertiary Education	22,001 (8.1)	31,478 (11)		19,367 (8.5)	19,394 (8.5)	
Elementary	5,991 (2.2)	5,689 (1.9)		4,660 (2.0)	4,663 (2.0)	
Missing	78,135 (29)	96,104 (32)		70,026 (31)	70,030 (31)	
Ethnic group			0.089			0.057
Christian Arab	4,877 (1.8)	3,749 (1.3)		4,229 (1.9)	2,851 (1.2)	
Druse	6,177 (2.3)	5,504 (1.8)		4,970 (2.2)	4,922 (2.2)	
Jewish	153,066 (56)	168,623 (57)		124,390 (55)	123,310 (54)	
Muslim Arab	65,060 (24)	69,204 (23)		57,896 (25)	58,049 (25)	
Muslim Bedouin	4,534 (1.7)	2,974 (1.0)		2,903 (1.3)	2,799 (1.2)	

© 2025 Goldshtein I et al. *JAMA Network Open*.

	Pre-Matching, Children, No. (%)			Post-Matching, Children, No. (%)		
	Less than 6 months of breastfeeding	Breastfeeding for at least 6 months		Less than 6 months of breastfeeding	Breastfeeding for at least 6 months	
**Characteristic, n (%)**	**N = 272,961**	**N = 297,571**	**SMD**	**N = 228,210**	**N=228,210**	**SMD**
Other	5,549 (2.0)	6,202 (2.1)		4,112 (1.8)	4,121 (1.8)	
Missing	33,698 (12)	41,315 (14)		29,710 (13)	32,158 (14)	
Sex			0.007			0.002
Female	132,771 (49)	145,808 (49)		111,532 (49)	111,272 (49)	
Male	140,190 (51)	151,763 (51)		116,678 (51)	116,938 (51)	
Maternal birth country			0.11			0.047
Israel	221,478 (81)	236,860 (80)		185,930 (81)	183,303 (80)	
Former Soviet Union	16,865 (6.2)	15,604 (5.2)		12,241 (5.4)	12,215 (5.4)	
Europe	3,960 (1.5)	4,442 (1.5)		3,428 (1.5)	3,242 (1.4)	
America	2,056 (0.8)	5,515 (1.9)		1,786 (0.8)	1,814 (0.8)	
Ethiopia	3,913 (1.4)	4,280 (1.4)		2,973 (1.3)	3,643 (1.6)	
Other	1,528 (0.6)	2,246 (0.8)		1,206 (0.5)	1,764 (0.8)	
Missing	23,161 (8.5)	28,624 (9.6)		20,646 (9.0)	22,229 (9.7)	
Maternal employment			0.047			0.034
Working	120,963 (44)	130,181 (44)		98,788 (43)	95,548 (42)	
Not Working	58,511 (21)	60,208 (20)		47,784 (21)	50,511 (22)	
Student	10,833 (4.0)	13,800 (4.6)		9,821 (4.3)	10,116 (4.4)	
Missing	82,654 (30)	93,382 (31)		71,817 (31)	72,035 (32)	
Socioeconomic status (1-10), Median (IQR)	5 (3, 6)	4 (3, 6)	0.10	5 (3, 6)	4 (3, 6)	0.056
Any comorbidity	61,042 (22)	55,851 (19)	0.089	48,668 (21)	43,002 (19)	0.062
Any bio-social risk	47,566 (17)	34,558 (12)	0.2	31,377 (14)	31,138 (14)	0.003
Any vision disorder	14,678 (5.4)	13,821 (4.6)	0.034	12,241 (5.4)	12,570 (5.5)	0.006
Marital status			0.11			0.034
Married	232,337 (85)	257,571 (87)		197,172 (86)	197,173 (86)	

© 2025 Goldshtein I et al. *JAMA Network Open*.

	Pre-Matching, Children, No. (%)			Post-Matching, Children, No. (%)		
	Less than 6 months of breastfeeding	Breastfeeding for at least 6 months		Less than 6 months of breastfeeding	Breastfeeding for at least 6 months	
**Characteristic, n (%)**	**N = 272,961**	**N = 297,571**	**SMD**	**N = 228,210**	**N=228,210**	**SMD**
Non-married	17,907 (6.6)	12,151 (4.1)		10,926 (4.8)	9,596 (4.2)	
Missing	22,717 (8.3)	27,849 (9.4)		20,112 (8.8)	21,441 (9.4)	
Maternal age group, years			0.050			0.06
<=20	1,201 (0.4)	897 (0.3)		973 (0.4)	681 (0.3)	
21-40	237,801 (87)	256,022 (86)		199,543 (87)	195,473 (86)	
>40	24,633 (9.0)	28,080 (9.4)		19,280 (8.4)	22,197 (9.7)	
Missing	9,326 (3.4)	12,572 (4.2)		8,414 (3.7)	9,859 (4.3)	
Maternal age, years	29 (25-33)	29 (25-34)	0.048	29 (25-33)	29 (25-33)	0.056
Newborn Position			0.089			0.018
Head	226,670 (83)	254,913 (86)		192,685 (84)	191,749 (84)	
Breech	9,579 (3.5)	6,686 (2.2)		5,862 (2.6)	6,289 (2.8)	
Other	2,898 (1.1)	2,637 (0.9)		1,981 (0.9)	2,260 (1.0)	
Missing	33,814 (12)	33,335 (11)		27,682 (12)	27,912 (12)	
Birth type			0.2			0.001
Spontaneous	188,863 (69)	232,710 (78)		169,187 (74)	169,317 (74)	
Caesarean section	53,012 (19)	36,489 (12)		34,538 (15)	34,460 (15)	
Instrumental	15,965 (5.8)	14,726 (4.9)		12,260 (5.4)	12,242 (5.4)	
Missing	15,121 (5.5)	13,646 (4.6)		12,225 (5.4)	12,191 (5.3)	
EPDS score (postpartum screening scale)			0.069			0.034
<10	197,135 (72)	222,846 (75)		166,858 (73)	166,060 (73)	
10+	9,230 (3.4)	7,534 (2.5)		7,303 (3.2)	6,164 (2.7)	
(Missing)	66,596 (24)	67,191 (23)		54,049 (24)	55,986 (25)	
Birth year	2017 (2015, 2019)	2017 (2015, 2019)	0.007	2017 (2015, 2019)	2016 (2015, 2018)	0.2
Apgar 1 minute			0.032			0.011

© 2025 Goldshtein I et al. *JAMA Network Open*.

	Pre-Matching, Children, No. (%)			Post-Matching, Children, No. (%)		
	**Less than 6 months of breastfeeding**	**Breastfeeding for at least 6 months**		**Less than 6 months of breastfeeding**	**Breastfeeding for at least 6 months**	
**Characteristic, n (%)**	**N = 272,961**	**N = 297,571**	**SMD**	**N = 228,210**	**N=228,210**	**SMD**
0-3	264,695 (97)	289,695 (97)		221,734 (97)	222,059 (97)	
4-6	1,128 (0.4)	980 (0.3)		836 (0.4)	857 (0.4)	
7-10	3,907 (1.4)	3,304 (1.1)		2,911 (1.3)	2,772 (1.2)	
Missing	3,238 (1.2)	3,585 (1.2)		2,736 (1.2)	2,515 (1.1)	
Apgar 5 minutes			0.007			0.014
0-3	267,219 (98)	291,307 (98)		223,404 (98)	223,653 (98)	
4-6	443 (0.2)	500 (0.2)		358 (0.2)	431 (0.2)	
7-10	415 (0.2)	376 (0.1)		304 (0.1)	315 (0.1)	
Missing	4,891 (1.8)	5,381 (1.8)		4,151 (1.8)	3,804 (1.7)	

© 2025 Goldshtein I et al. *JAMA Network Open*.

eTable 4. Population characteristics, siblings' analysis

	Median (IQR)		
	**Sibling with less than 6 months of breastfeeding**	**Sibling with breastfeeding for at least 6 months**	
**Characteristic, n (%)**	**N = 37,704**	**N = 37,704**	**SMD**
Breastfeeding duration, months	2 (0 to 4)	10 (7 to 14)	1.76
Multiple pregnancy	812 (2.3)	442 (1.3)	0.08
Gestational age, weeks	39 (38 to 40)	39 (38 to 40)	0.06
Small for gestational age	2,954 (7.9)	2,453 (6.5)	0.05
Firstborn, n (%)	17,994 (48)	15,231 (40)	0.15
Sex			0.004
Female	18,560 (49)	18,640 (49)	
Male	19,144 (51)	19,064 (51)	
Maternal age, years	28 (24 to 32)	28 (24 to 32)	0.04
Newborn Position			0.12
Head	30,806 (82)	32,349 (86)	
Breech	903 (2.4)	812 (2.2)	
Other	338 (0.9)	341 (0.9)	
Missing	5,567 (15)	4,202 (11)	
Birth type			0.16
Spontaneous	28,082 (74)	29,762 (79)	
Caesarean section	4,855 (13)	4,649 (12)	
Instrumental	1,851 (4.9)	1,704 (4.5)	
Missing	2,916 (7.7)	1,589 (4.2)	
Birth year	2017 (2015-2018)	2017 (2015-2019)	0.1
Apgar 1 minute			0.07
0-3	219 (0.6)	155 (0.4)	
4-6	484 (1.3)	439 (1.2)	

© 2025 Goldshtein I et al. *JAMA Network Open*.

	Median (IQR)		
	Sibling with less than 6 months of breastfeeding	Sibling with breastfeeding for at least 6 months	
**Characteristic, n (%)**	**N = 37,704**	**N = 37,704**	**SMD**
7-10	36110 (96)	36538 (97)	
Missing	891 (2.4)	572 (1.5)	
Apgar 5 minutes			0.06
0-3	124 (0.3)	94 (0.2)	
4-6	67 (0.2)	70 (0.2)	
7-10	36,366 (96)	36,724 (97)	
Missing	1,147 (3.0)	816 (2.2)	

© 2025 Goldshtein I et al. *JAMA Network Open*.

eFigure 1. Study population flow chart

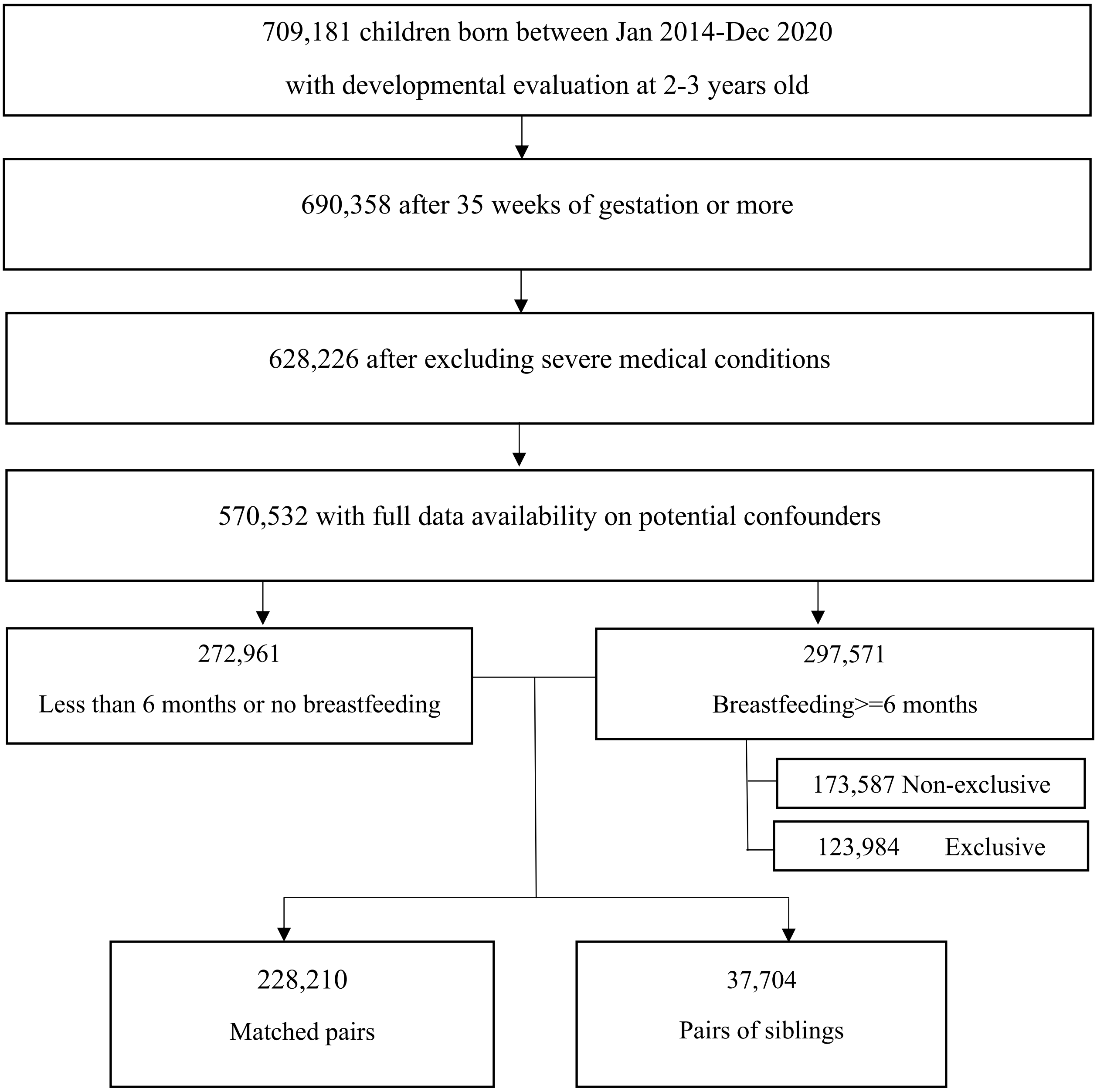

© 2025 Goldshtein I et al. *JAMA Network Open*.

eFigure 2. Histogram of breastfeeding duration (months)

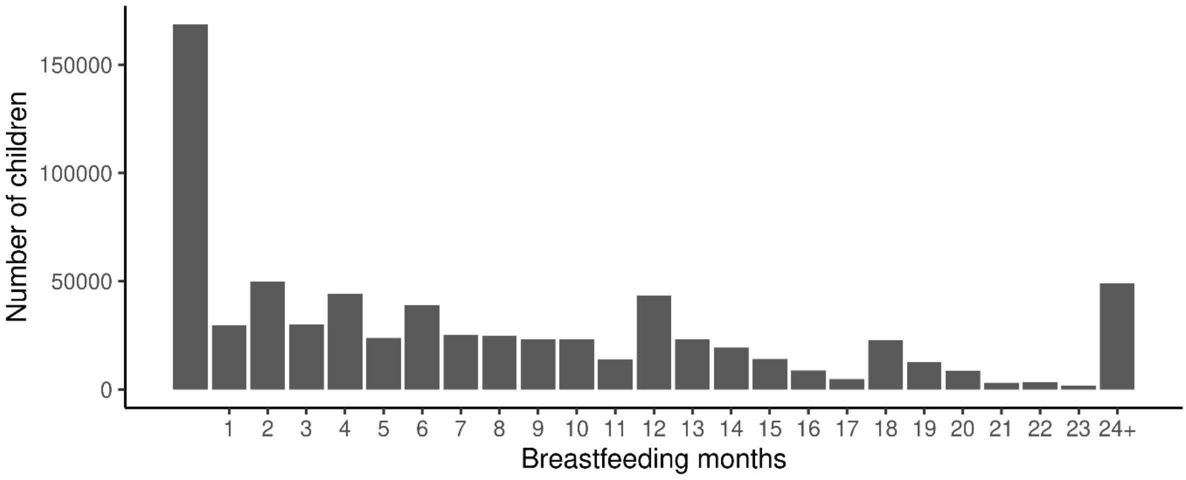

© 2025 Goldshtein I et al. *JAMA Network Open*.

eFigure 3. Restricted cubic spline for the absolute probability of developmental outcomes as a function of breastfeeding duration

Figure 3.a Overall delay/neurodevelopmental condition

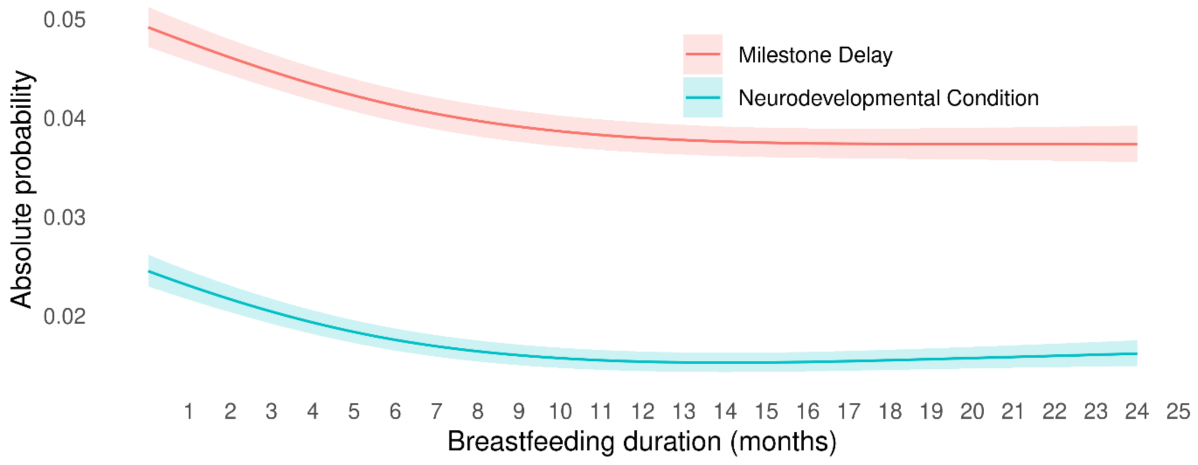

Figure 3.b Language social delay/ neurodevelopmental condition

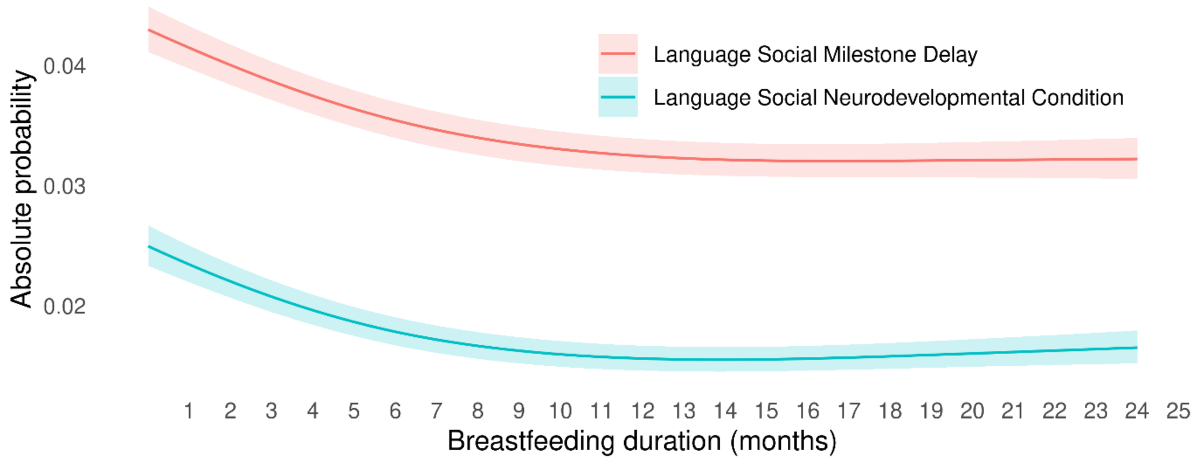

© 2025 Goldshtein I et al. *JAMA Network Open*.

Figure 3.c Motor delay/ neurodevelopmental condition

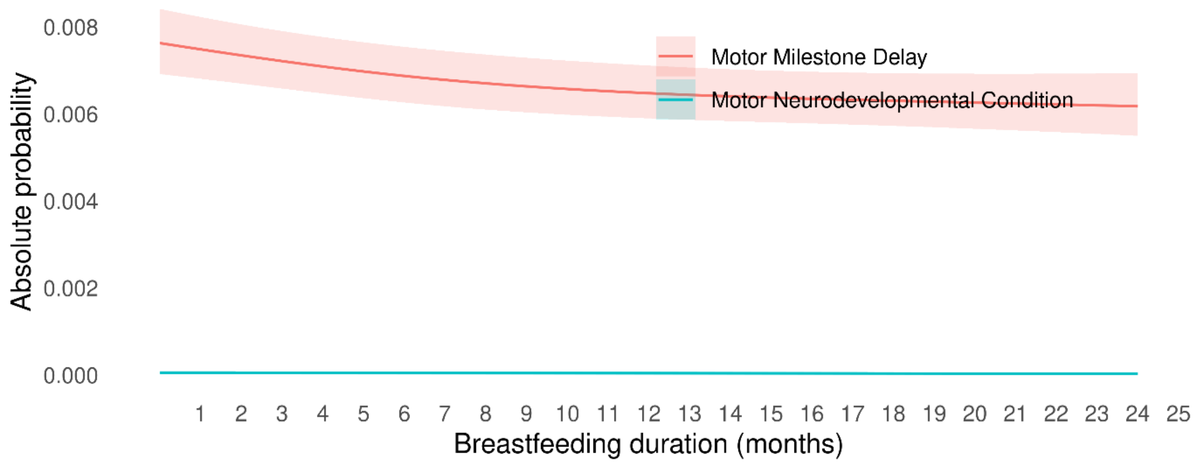

Absolute probability of developmental outcomes by exposure to breastfeeding duration, calculated using the mode value of maternal, birth and infant-related factors. Shading indicates 95% CIs.

© 2025 Goldshtein I et al. *JAMA Network Open*.
