# Supplementary material for: Breastfeeding Duration and Child Development
Source: JAMA Netw Open. 2025 Mar 24;8(3):e251540. doi: 10.1001/jamanetworkopen.2025.1540 (PMC11933992; doi:10.1001/jamanetworkopen.2025.1540)
Supplement: Supplement 2. — Data Sharing Statement [file jamanetwopen-e251540-s002.pdf]

Data Sharing Statement

Goldshtein. Breastfeeding Duration and Child Development. *JAMA Netw Open*. Published March 24, 2025. doi:10.1001/jamanetworkopen.2025.1540

Data
Data available: No
